# The CombiConsultation: a new concept of sequential consultation with the pharmacist and practice nurse/general practitioner for patients with a chronic condition.

**DOI:** 10.1007/s11096-021-01350-y

**Published:** 2021-11-10

**Authors:** Valérie A. M. Meijvis, Mette Heringa, Henk-Frans Kwint, Niek J. de Wit, Marcel L. Bouvy

**Affiliations:** 1 SIR Institute for Pharmacy Practice and Policy, 2331 JE Leiden, The Netherlands; 2 Department of Pharmaceutical Sciences, Utrecht University, Utrecht, The Netherlands; 3 Department of General Practice, Julius Centre for Health Sciences and Primary Care, University Medical Centre Utrecht, Utrecht, The Netherlands

**Keywords:** CombiConsultation, Community pharmacist, General practitioner, Pharmaceutical care, Practice nurse, Primary care

## Abstract

The primary health care system is generally well organized for dealing with chronic diseases, but comprehensive medication management is still a challenge. Studies suggest that pharmacists can contribute to effective and safe drug therapy by providing services like a clinical medication review (CMR). However, several factors limit the potential impact of a CMR. Therefore, we propose a new pharmaceutical care service for patients with a chronic condition: the CombiConsultation. The CombiConsultation is a medication evaluation service conducted by the (community) pharmacist and either the practice nurse or general practitioner. It consists of 3 steps: medication check, implementation and follow-up. The pharmacist primarily focusses on setting treatment goals for 1 or 2 drug-related problems in relation to a specific chronic condition. In this manuscript we describe the process and characteristics of the CombiConsultation. We compare the CombiConsultation with the CMR and explain the choices made and the implications for implementation.

## Introduction

## Background

Aging and the related increase in chronic conditions and multi‐morbidity lead to an increased demand for care. In the Netherlands, the number of people with at least 1 chronic condition is predicted to increase from 8.5 million (of 17 million) in 2015 to 9.8 million in 2040 [1].

Chronic diseases require appropriate management. The provision of care for most chronic diseases has shifted from secondary to primary care, mainly for cost effectiveness reasons [2]. This has led to the development of new models for primary care for patients with chronic conditions, which share an integrated, patient-centred and pro-active approach [3]. Due to the increasing workload of general practitioners (GPs), the role of physician assistants and practice nurses (PNs) has become more important [4]. In the Netherlands, PN’s provide chronic care to patients with Diabetes Mellitus (DM), chronic obstructive pulmonary disease (COPD) and (risk of) cardiovascular problems. Although the primary health care system is generally well organized for addressing chronic diseases, comprehensive medication management is still a challenge. Studies suggest that pharmacists may contribute to effective and safe drug therapy by providing clinical pharmacy services [5]. In the Netherlands, community pharmacists are actively involved in patient care and they are experienced in performing Clinical medication reviews (CMRs) together with GPs. Nevertheless, there are significant barriers to community pharmacists to implementing clinical services, including lack of mandate, effectiveness and readiness to embrace change [6].

CMRs are among the most studied and effective interventions performed by pharmacists. A CMR is a structured, critical examination of patient’s drug therapy with the objective of optimising the beneficial effects of medicines, minimising the number of drug-related problems (DRPs) and increasing the efficiency of pharmacotherapy. A CMR consists of 5 steps: 1. Patient interview, 2. Analysis: identifying DRPs, 3. Discussion GP and pharmacist, 4. Implementation of actions, 5. Follow-up and monitoring [7]. In current practice, several factors limit the potential impact of CMR for patients with chronic conditions.

First, most pharmacists do not have the resources to offer a CMR to all patients with chronic conditions. Therefore, additional selection criteria (like higher age, number of medicines or frailty scores) are generally used to identify patients eligible for a CMR. In the Netherlands, the selection criteria of ≥ 65 years old and ≥ 5 medicines in use have recently been adjusted to ≥ 75 years old and ≥ 10 medicines in use (and/or frailty) to keep the number of eligible patients manageable. However, a medication review can be relevant for all patients with chronic conditions requiring chronic drug use in order to optimize the effectiveness of prescriptions and limit the risk of drug use in the long term [8].

Second, the implementation rate of recommendations can often be improved. Kwint et al. demonstrated that implementation rates of recommendations resulting from medication reviews vary from 17 to 86%. The implementation rate was strongly associated with the extent of collaboration between pharmacists and GPs [9].

There is a need to address the limitations in feasibility and efficiency of the model of CMR. Thus, in this manuscript we propose a new pharmaceutical care service for patients with chronic conditions, the CombiConsultation, and we describe its design and features.

## Design of the concept

**Fig. 1:**
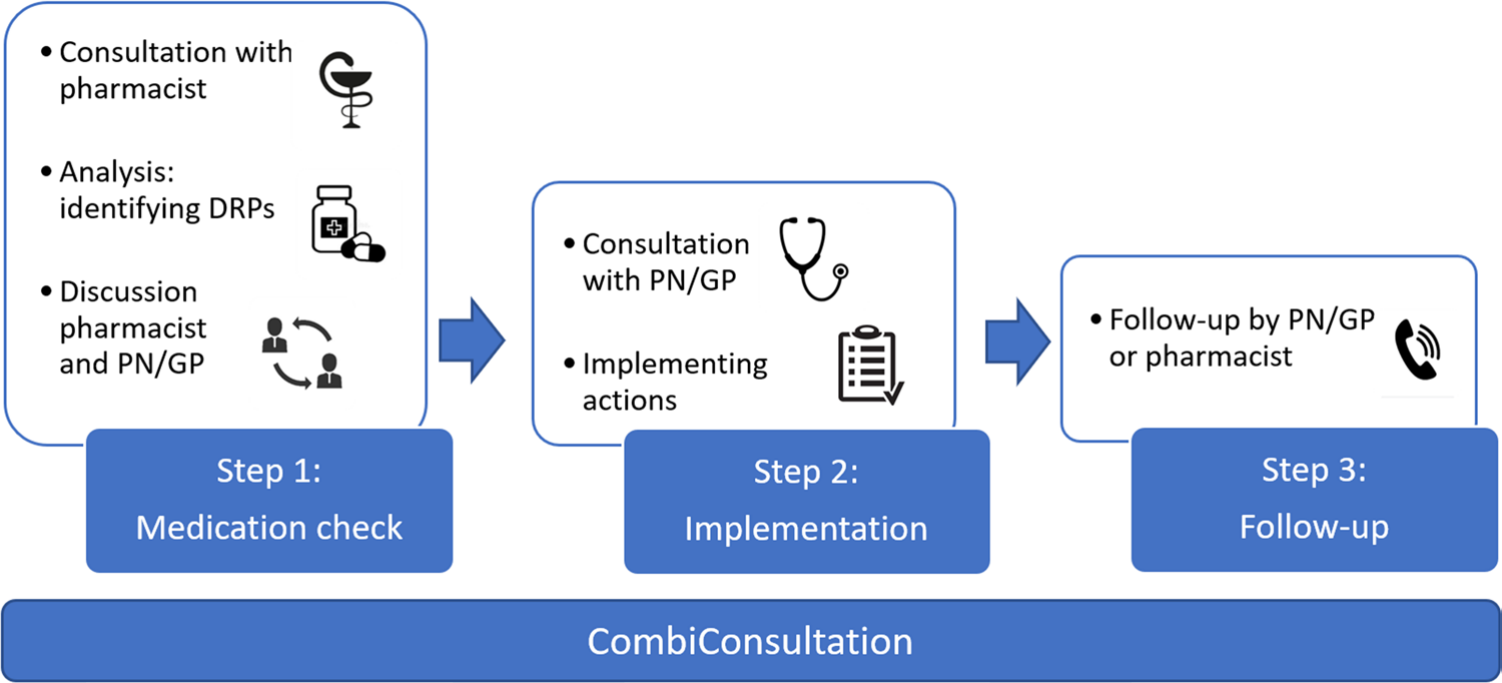
Schematic representation of the 3 steps of the CombiConsultation. *PN* practice nurse, *GP* general practitioner, *DRPs* drug related problems

Comparable with a CMR (or medication review type 3), a CombiConsultation is based on medication history, patient information and clinical information [10]. However, in contrast to a full CMR the CombiConsultation focuses on the medication for a specific condition. The CombiConsultation is conducted by the (community) pharmacist and either the PN and/or GP. The patient visits the PN and/or GP immediately after the consultation with the pharmacist. We describe the process first (Fig. 1) and then the characteristics of the CombiConsultation (Table 1).

**Table 1:** Characteristics of the CMR and CombiConsultation

Characteristic	Traditional CMR*	CombiConsultation
Target population	65+ ≥ 5 medicines	18+ ≥ 1 medicine Patients with any chronic condition that requires chronic drug treatment
Aim	Complete medical history and drug history	1–2 health-related complaints in relation to the chronic condition
Duration of patient consultation with pharmacist	30–50 min	15–20 min
Setting	Pharmacy, patient’s home, or at the general practice	General practice

*Stepwise approach of a CMR according to multidisciplinary guideline ‘Polypharmacy in the Elderly’: 1. Patient interview, 2. Analysis: identifying DRPs, 3. Discussion between GP and pharmacist, 4. Implementation of actions, 5. Follow-up and monitoring

### Process

The CombiConsultation consists of 3 steps (Fig. 1).

- *Step 1* Medication Check.

- Consultation with the pharmacist
  The patient first receives a medication consultation of 15–20 minutes with the pharmacist shortly before a consultation with the PN/GP concerning the chronic condition (Step 2). The pharmacist has access to the medication history and clinical information, like diagnoses and laboratory values. The focus of the pharmacist during the consultation is to identify 1 or 2 main health-related complaints in relation to the chronic condition. If there are several problems, the pharmacist and patient decide together which problem(s) has the highest priority. They set 1 or 2 specific treatment goals.
- Analysis: identifying DRPs
  The pharmacist identifies DRPs based on the consultation as well as clinical information and medication history. Based on the identified DRPs and treatment goals, the pharmacist summarises the recommendations for action in a short pharmaceutical care plan.
- Discussion

       The pharmacist discusses the pharmaceutical care plan with the PN/GP. Follow-up times are scheduled.

- *Step 2* Implementation.

- Consultation with PN/GP
  The patient next consults with the PN/GP concerning their chronic condition.
- Implementing actions

       During the consultation with the PN/GP, the pharmaceutical care plan is discussed with the patient, and actions are implemented.

- *Step 3* Follow-up by the pharmacist or PN/GP.

- Follow-up by pharmacist or PN/GP

       Two to 4 weeks after the initial medication consultation, the pharmacist or PN/GP (depending on the agreement made in Step 1) has a follow-up consultation with the patient to evaluate the implemented actions. Monitoring is then continued for as long as necessary.

### Characteristics

Table 1 compares the CombiConsultation with the CMR. We explain the choices made and the implications for implementation.

#### Target population

The CombiConsultation has been developed for adult patients with at least 1 chronic condition that requires ongoing drug treatment. This contrasts with the usual selection criteria for a CMR, which generally consist of a combination of higher age, polypharmacy, multimorbidity or frailty. However, all adult patients with a single chronic condition may experience problems with medication. Therefore, all patients who use at least 1 medication and receive primary care treatment for a chronic condition are eligible for a CombiConsultation.

#### Aim

The CombiConsultation focusses primarily on setting treatment goals for 1 or 2 health-related complaints in relation to a specific chronic condition (e.g., DM or COPD). In contrast, the CMR provides a full analysis of potential DRPs such as deviations from guidelines and inappropriate prescriptions. Several studies recommend shifting the focus of CMR to issues that the patient perceives as most relevant [11]. It has been demonstrated that specific attention to patients’ individual health goals along with follow-up and monitoring of the suggested interventions leads to a higher implementation rate [12]. When the patient has multiple health related complaints, the pharmacist and patient agree which problem has the priority or agree to plan a CMR. The pharmacist primarily focusses on the medication for the chronic condition; however, it is also possible to discuss medication for other conditions if the patient wishes.

#### Duration of the patient consultation with pharmacist

In contrast with a CMR, a CombiConsultation targets less complex patients and specifically focusses on 1 or 2 problems. Based on an unpublished pilot study in 96 patients (analysis not included), a consultation of 15–20 min is expected to be appropriate. Previous research has shown that a CMR patient interview took 35–50 min [13].

#### Setting

The medication check of the CombiConsultation is preferentially conducted by the pharmacist in the GP’s practice, which emphasizes the cooperation with the PN/GP and allows for more direct communication between the pharmacist and PN/GP. It is not a requirement that the pharmacist is working in the GP practice as long as the pharmacist has access to clinical information. Integrating pharmacists into the patient care team within primary care practices leads to better patient outcomes and medication use [14]. Moreover, this integration can improve the acceptance of drug-related recommendations and optimise pharmacotherapy and drug safety. Studies assessing the impact of non-dispensing pharmacists working in a general practice have demonstrated the identification of a high number of DRPs and higher implementation rates during the process of CMR [14]. Besides, pharmacists can make better decisions when they have access to medical information from the GP upon which they can base their decisions [15].

As mentioned above, in a CombiConsultation the consultation with the pharmacist is directly followed by the consultation with the PN/GP: a one-stop-shop approach. Immediately after the consultation, the pharmacist communicates (in person or electronically) the 1 or 2 main recommendations to the PN/GP. Therefore, interventions can be implemented directly during the PN/GP consultation. Also, the patient can go directly to the next healthcare provider. This may lead to better perceived quality of care, especially in terms of accessibility and continuity of care [16].

## Discussion

We suggest the CombiConsultation as an alternative to improve pharmaceutical care for patients with chronic disease in primary care. By assessing their problems and concerns related to medication and by using shared decision-making to set personal treatment goals, patients become more involved in their own treatment. This is particularly important with chronic conditions to prevent complications over the long term [12]. A systematic review by Raynolds indicates that self-management support (improving the participation and self-reliance of the patient) most frequently results in improvements in patient–level outcomes, predominately for diabetes and hypertension [17].

Because of its limiting selection criteria, the CMR excludes a large group of patients, resulting in a need for other types of medication evaluation. In the Netherlands, the guideline for medication review recommend targeted medication consultations, such as evaluation of correct use of medication, evaluation of a specific medication-related health problem or evaluation of the medication for a specific condition, like the CombiConsultation [18]. By focusing on patients with at least 1 chronic condition and 1 prescription (irrespective of age), it is possible to increase the target group. If there is not enough time for adequate discussion during the CombiConsultation, the pharmacist can invite the patient for a CMR; thus, the CombiConsultation can be used as a pre-selection tool for the CMR.

As mentioned before, in 2019 the selection criteria for a CMR in the Netherlands have been adjusted to select candidates most likely to benefit from a CMR. To prevent that implementation of the CombiConsultation will lead to numbers that exceed the pharmacy workforce capacity, it is desirable to start with a specific patient group, for example DM. In the Netherlands, patient groups with a single chronic condition (e.g. Diabetes Mellitus) are already monitored by the PN. This makes it easier to implement the CombiConsultation focused on these specific patient groups. Nevertheless, it is certainly feasible to select a patient group in other settings.

To perform the CombiConsultation properly, a basic level of interprofessional collaboration is required. Clear agreements about patient selection, planning, inviting patients and practical implementation are necessary. In the proposed process, the patient consults with the pharmacist before the PN/GP. However, in daily practice the reverse order could be considered. A potential advantage of the reverse order is the availability to the pharmacist of recent clinical data such as blood pressure following the PN/GP check-up. However, a significant disadvantage is that recommendations from the medication check cannot then be immediately implemented with the PN/GP, and the patient may still need to be informed about additional interventions after the CombiConsultation. The pharmacist has the expertise to perform the medication check. Further investigation is needed to explore the potential role of other healthcare providers (like pharmacy technicians) in the CombiConsultation.

As with a CMR, the pharmacist must be professionally trained to perform a CombiConsultation. Many health care providers, including pharmacists, offer patients advice and information about their medicine. However, when providers focus on identifying the patient’s needs and concerns about medication, they are more likely to address the problems most relevant to the patient [19]. Historically, little attention was given to consultation skills in pre-graduate pharmacist training. Although training in consultation skills is more common today, some pharmacists, especially those who are older, may need additional training in patient-centred communication. Other important skills that may require training are clinical reasoning and shared decision making [20].

Before the CombiConsultation can be implemented on a large scale, it is necessary to demonstrate the added value of the intervention. Research is needed to assess which patients may benefit the most as well as to evaluate the experience of healthcare providers and patients regarding implementation barriers and facilitators. An intervention study is currently conducted.

## Conclusion

The CombiConsultation is a new approach to improve the outcomes of pharmacotherapy in patients with a chronic condition by providing a medication evaluation service conducted in close collaboration between pharmacist and PN/GP. The concept relies on pharmacists to deliver patient-centred care, which requires consultation skills and the ability to cooperate with other care providers. Research is needed to evaluate the feasibility and possible effects of the CombiConsultation.
